# Relationship between stunting in children 6 to 36 months of age and maternal employment status in Peru: A sub-analysis of the Peruvian Demographic and Health Survey

**DOI:** 10.1371/journal.pone.0212164

**Published:** 2019-04-03

**Authors:** Airin Chávez-Zárate, Jorge L. Maguiña, Antoinette Danciana Quichiz-Lara, Patricia Edith Zapata-Fajardo, Percy Mayta-Tristán

**Affiliations:** 1 Escuela de Nutrición y Dietética, Universidad Peruana de Ciencias Aplicadas, Lima, Perú; 2 Escuela de Medicina, Universidad Peruana de Ciencias Aplicadas, Lima Perú; Tulane University School of Public Health and Tropical Medicine, UNITED STATES

## Abstract

**Objectives:**

This study aimed to determine the relationship between stunting in children 6 to 36 months old and maternal employment status in Peru.

**Methods:**

A secondary data analysis was conducted using information from the Demographic and Health Survey (DHS) in Peru. We used a representative sample of 4637 mother-child binomials to determine the association between stunting in children 6 to 36 months of age and the employment status of their mothers.

**Results:**

The prevalence of stunting among children was 15.9% (95% CI: 13.9–16.7). The prevalence of working mothers was 63.7%. No association was found between maternal employment status and the presence of stunting in children [prevalence ratio (PR) = 1.04; 95% confidence interval (95% CI): 0.9 to 1.2; p = 0.627). However, on multivariate analysis we found that the prevalence of stunting was significantly higher among children of mothers performing unpaid work (12.4%) (PR = 1.38; 95% CI: 1.2–1.6; p < 0.001) compared with those of paid working mothers.

**Conclusion:**

No significant association was found between maternal employment status and the presence of stunting in children 6 to 36 months of age. However, children of mothers doing unpaid work are at higher risk of stunting. These findings support the implementation of educational programs and labour policies to reduce the prevalence of stunting among children.

## Introduction

Child nutritional status, especially undernutrition, is still an important public health problem, specifically in developing countries[1–5]. The last publication of *The State of World’s Children* reported that 25.0% of children under 5 years old are stunted worldwide[1]. In Latin America, the average frequency of stunting was 6.6% in 2016[1,5], while in Peru the prevalence of stunting in children under 5 years of age was 14.6%[6], being above the Latin American average. This prevalence suggests that stunting is one of the main nutritional problems to combat in our country[7–8].

Inadequate dietary intake is not the only underlying cause of stunting in children (UNICEF-2013)[1–2,6]. It has been suggested that maternal employment status plays a critical role, possibly due to early initiation of complementary feeding [9–10]. Economic, social, and cultural changes have led to increased participation of women in the labour market, which can indirectly influence the development and growth of their children[11]. In the last ten years, the proportion of working women in Peru has increased from 58.0% to 68.4%[12–13].

The association between child stunting and the employment status of mothers is controversial. Some studies have reported that the presence of stunting in children is higher when the mother is working[14–16]. By contrast, other studies in Asian[17–18] and Latin American countries[19–20] show that the prevalence of stunting decreases with working mothers.

In these studies, in addition to having a higher prevalence of stunted children in common, these mothers also belong to families from rural areas of developing countries with low economic income and a low wealth index. Some examples of unpaid labour include working for family members or neighbours taking care of livestock and crops.

Although the majority of mothers in Peru are active workers[12–13], no study has evaluated the potential association between child stunting and maternal employment status.

## Methods

**Fig 1:**
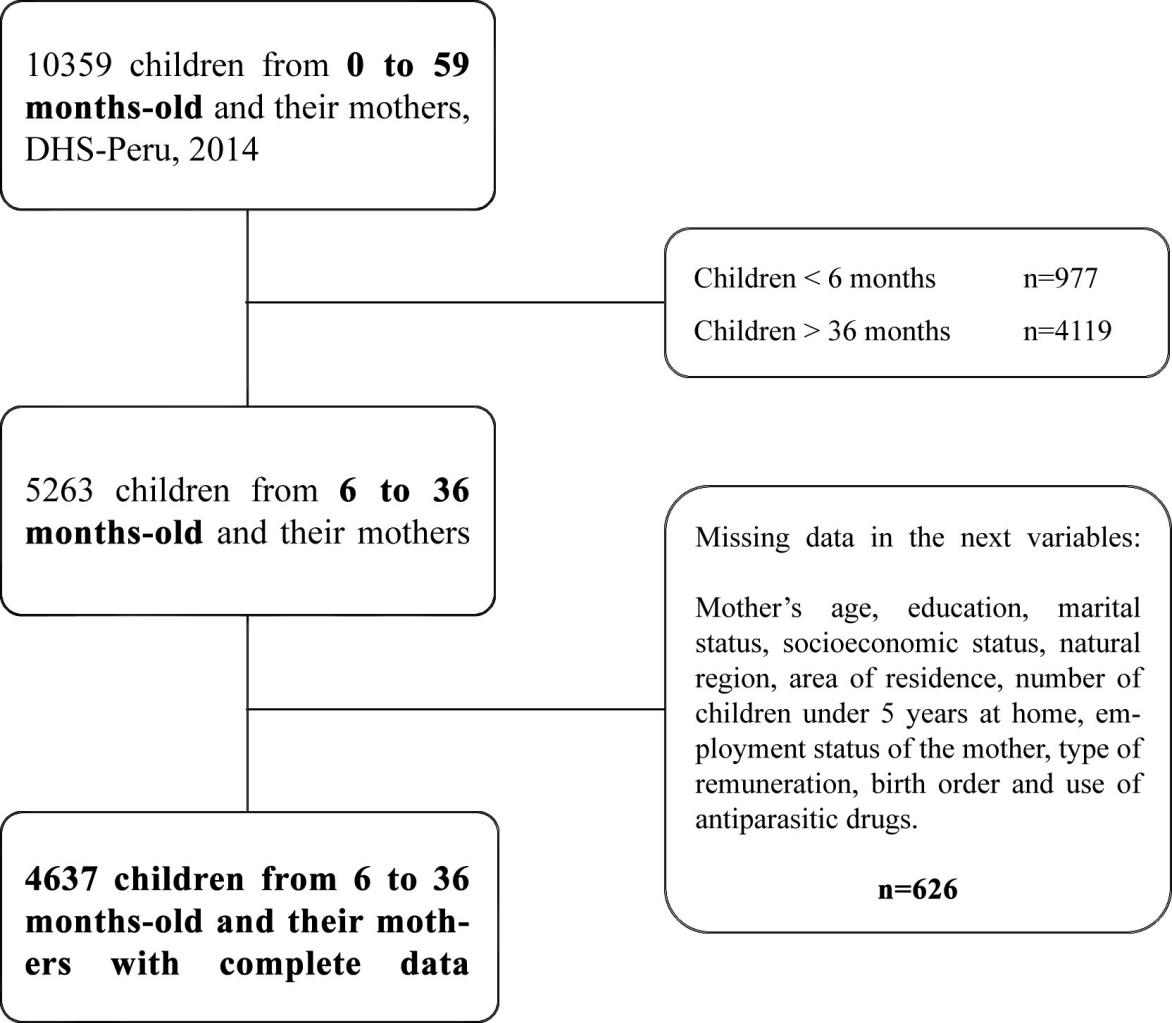
Exclusion flow chart of the population from the DHS-Peru 2014 for the study sample.

We conducted a secondary analysis using data from the Demographic Health Survey in Peru (DHS-Peru) collected by the Instituto Nacional de Estadística e Informática (INEI) in 2014. This was a probabilistic, stratified, two-stage, independent, and self-weighted survey conducted by a department that collected information from 29,941 households. For the present study, we used a representative sample for analysis to provide indicators of nutritional status of children. Each child was used as a primary sampling unit in order to analyse all possible mother-child binomials. Participants lacking data on exposure, outcome or control variables were not included in the analysis (Fig 1).

### Outcome variables

According to the World Health Organization (WHO) stunting is defined as a height for age (H/A) value less than 2 standard deviations (SD) of the WHO Child Growth Standards median[21]. The measurement of children’s length and height from the DHS-Peru was performed by trained personnel[22].

### Exposure variables

We generated two outcome variables: First, we categorized mothers who had not worked for 12 months prior to the survey as “non-working” and those working at the time of the survey or during the previous 12 months as “working”. Women exclusively dedicated to homemaking were considered as "non-working". Second, the variable remuneration was categorized as: paid in money and/or in-kind, unpaid and non-working mothers.

### Co-variables of interest

We analysed the sociodemographic variables of mothers including: age (15 to 24 years, 25 to 34 years, and 35 to 49 years), level of education (without education, primary, secondary, superior), marital status (with or without partner), economic status (very poor/poor or medium/rich/very rich), region of residence (coast, Andes highland, amazon basin), area of residence (rural or urban), and number of children under 5 years old (1, 2, 3 or more). We also analysed the characteristics of the children including: current age in months (6 to 12 months, 13 to 18 months, 19 to 24 months, 25 to 30 months, from 31 to 36 months), gender (male or female), birth order (1^st^ child, 2^nd^– 4^th^, 5^th^ or more) and use of antiparasitic drugs (no or yes). Finally, in order to more accurately describe how extreme poverty is significantly associated with unpaid work and stunting, we used the original Wealth Index of the DHS-Peru that consists in five levels (very poor, poor, medium, rich, very rich) for the association between the type of remuneration of working mothers and the presence of stunting in children from 6 to 36 months of age.

### Power analysis

For the secondary analysis 4637 records with complete data from the DHS-Peru were available, with a power ≥ 80.0% to detect a prevalence ratio ≥1.2, assuming a prevalence of stunted children from working women of 27.0% and an r2 of 0.2[19]. The analysis was performed using the PASS 11.0 program (NCSS, LLC. Kaysville, Utah, USA).

### Data analysis

The analyses were performed using Stata 14, statistical software for Windows (StataCorp, College Station, TX, US). We took into account the survey design of the study as sample strata, primary sampling units and population weights[6]. The Chi-squared test was used to determine the association between categorical variables. Bivariate and multivariate analysis for our two outcome variables were conducted using Poisson regression models to calculate the unadjusted prevalence ratio (PR(u)) and the adjusted prevalence ratio (PR(a)), respectively. All statistical analyses were conducted with 95% confidence interval and a level of significance less than 0.05.

### Ethical standards disclosure

The Institutional Research Board (IRB) of the Universidad Peruana de Ciencias Aplicadas (CEI/UPC 681 PI278) approved the study. The databases are available on the website of INEI (http://iinei.inei.gob.pe/microdatos/). These databases are anonymous and do not allow the identification of participants.

## Results

### Characteristics of the study population

The survey included a representative sample of 4,637 children from 6 to 36 months of age and their mothers. (Fig 1)

Tables 1 and 2 show the characteristics of the mothers between 15 to 49 years old. Only 61.1% had completed secondary education, 86.4% lived with their partners, 59.7% were poor or very poor, 37.5% inhabited the coastal region, and 59.8% lived in an urban setting. Regarding employment status, 63.7% were working mothers, and of this group, 53.4% were paid with money, whereas 12.4% did not receive any type of remuneration.

**Table 1:** Sociodemographic characteristics of mothers with children between 6 to 36 months of age according to their employment status (N = 4637).

Characteristics of mothers	Maternal employment				
	Work		No work		P-value
	(n 3051)		(n 1586)		
	n	(%)	n	(%)	
*Age*^*a*^					**<0.001**
15–24 years	823	(26.6)	588	(36.5)	
25–34 years	1429	(46.3)	683	(42.4)	
35–49 years	799	(27.1)	315	(21.1)	
*Level of education*^*a*^					**<0.001**
Without education	78	(1.9)	35	(2.0)	
Primary	830	(21.9)	414	(22.2)	
Secondary	1795	(63.6)	1039	(68.9)	
Superior	348	(12.6)	98	(6.9)	
*Marital status*^*a*^					**<0.001**
Without partner	498	(17.0)	122	(8.3)	
With partner	2553	(83.0)	1464	(91.7)	
*Economic level*^*a*^					0.158
Very poor / poor	1782	(45.4)	948	(48.3)	
Medium/ Rich / Very rich	1269	(54.6)	638	(51.7)	
*Region of Residence*^*a*^					**<0.005**
Coast	1067	(51.0)	698	(59.3)	
Andes highland	1190	(32.3)	468	(25.2)	
Amazon basin	794	(16.7)	420	(15.5)	
*Area of residence*^*a*^					**0.031**
Urban	1772	(70.3)	1036	(74.2)	
Rural	1279	(29.7)	550	(25.8)	
*Number of Children < 5 years*^*a*^					0.401
1	1855	(62.3)	901	(59.9)	
2	976	(31.0)	564	(33.7)	
3+	220	(6.7)	121	(6.4)	

^*a*^ Chi-squared test for categorical variables

**Table 2:** Sociodemographic characteristics of mothers with children 6 to 36 months of age according to the presence of stunting in children, taking into account the complex sample design (N = 4637).

Characteristics of mothers	Presence of stunting in children aged 6 to 36 months							
	Yes		No		P-value	Unadjusted model		
	(n 867)		(n 3770)					
	n	(%)	n	(%)		PR(u)	95% CI	P-value
*Age*^*a*^					0.513			
15–24 years	257	(14.8)	1154	(85.2)		Ref.		
25–34 years	375	(14.7)	1737	(85.3)		0.99	0.81–1.22	0.94
35–49 years	235	(16.5)	879	(83.5)		1.11	0.89–1.39	0.37
*Level of education*^*a*^					**<0.001**			
Without education	53	(49.2)	60	(50.8)		Ref.		
Primary	409	(30.8)	835	(69.2)		0.63	0.49–0.80	**<0.001**
Secondary	380	(10.6)	2454	(89.4)		0.22	0.17–0.28	**<0.001**
Superior	25	(4.8)	421	(95.2)		0.10	0.06–0.17	**<0.001**
*Marital Status*^*a*^					0.156			
Without partner	109	(13.0)	511	(87.0)		Ref.		
With partner	758	(15.6)	3259	(84.4)		1.20	0.93–1.55	0.162
*Economic level*^*a*^					**<0.001**			
Very poor / poor	724	(25.3)	2006	(74.7)		Ref.		
Medium/ Rich / Very rich	143	(6.5)	1764	(93.5)		0.26	0.20–0.33	**<0.001**
*Region of Residence*^*a*^					**<0.001**			
Coast	139	(6.9)	1626	(93.1)		Ref.		
Andes highland	452	(26.2)	1206	(73.8)		3.81	2.97–4.90	**<0.001**
Amazon basin	276	(22.8)	938	(77.2)		3.32	2.54–4.34	**<0.001**
*Area of residence*^*a*^					**<0.001**			
Urban	318	(9.3)	2490	(90.7)		Ref.		
Rural	549	(30.3)	1280	(69.7)		3.27	2.73–3.93	**<0.001**
*Number of children < 5 years of age*^*a*^					**<0.001**			
1	394	(11.0)	2362	(89.0)		Ref.		
2	372	(20.5)	1168	(79.5)		1.87	1.57–2.23	**<0.001**
3+	101	(28.9)	240	(71.1)		2.63	1.98–3.50	**<0.001**
*Employment status*^*a*^					0.166			
Not working	257	(14.0)	1329	(86.0)		Ref.		
Working	610	(15.9)	2441	(84.1)		1.14	0.95–1.37	0.167
*Type of remuneration*^*a*^					**<0.001**			
Paid in money and /or in-kind	390	(12.7)	2085	(87.3)		Ref.		
Unpaid	220	(37.3)	356	(62.7)		2.95	2.45–3.55	**<0.001**
Non-working	257	(14.0)	1329	(86.0)		1.10	0.91–1.34	0.322

^*a*^ Chi-squared test for categorical variables and Poisson regression in the unadjusted model with a 95% confidence interval taking into account the complex sample design: probabilistic, stratified, two-stage, independent, and self-weighted survey conducted by department.

Table 3 shows the characteristics of the children studied, with 22.6% being in the age group of 6 to 12 months, and 54.0% were the second, third or fourth child.

**Table 3:** Characteristics of children between 6 and 36 months of age according to the presence of stunting, taking into account the complex sample design (N = 4637).

Characteristics of the children	Presence of stunting in children aged 6 to 36 months							
	Yes		No		P-value	Unadjusted model		
	(n 867)		(n 3770)					
	n	(%)	n	(%)		PR(u)	95% CI	P-value
*Child age*^*a*^					**0.002**			
06–12 months	139	(11.5)	901	(88.5)		Ref.		
13–18 months	175	(15.8)	753	(84.2)		1.38	1.07–1.76	**0.014**
19–24 months	215	(18.7)	684	(81.3)		1.63	1.28–2.07	**<0.001**
25–30 months	158	(13.9)	716	(86.1)		1.21	0.92–1.60	0.174
31–36 months	180	(16.7)	716	(83.3)		1.45	1.13–1.87	**0.004**
*Gender*^*a*^					**0.002**			
Male	505	(17.0)	1880	(83.0)		Ref.		
Female	362	(13.3)	1890	(86.7)		0.78	0.66–0.91	**0.002**
*Birth order*^*a*^					**<0.001**			
First child	188	(9.2)	1326	(90.8)		Ref.		
2^nd^– 4^th^ child	460	(15.6)	2043	(84.4)		1.70	1.38–2.09	**<0.001**
5^th^ child or greater	219	(33.2)	401	(66.8)		3.61	2.86–4.55	**<0.001**
*Consumption of antiparasitic drugs in the last 6 months*^*a*^					**<0.001**			
No	585	(13.8)	2811	(86.2)		ref		
Yes	282	(19.7)	959	(80.3)		1.43	1.20–1.70	**<0**.**001**

^a^ Chi-squared test for categorical variables and Poisson regression in the unadjusted model with a 95% confidence interval taking into account the complex sample design: probabilistic, stratified, two-stage, independent, and self-weighted survey conducted by department.

### Prevalence of child stunting and associated factors

Among a representative sample of 4,637 children 6 to 36 months old, the prevalence of stunting was 15.9% (95% CI: 13.9–16.7). Likewise, unadjusted analysis showed that child stunting was associated with no education, very poor/poor economic status, residence in the Andes highland, residence in rural areas, having 3 or more children at home, no remuneration, children’s age ranging between 19 to 24 months, being males, being the fifth or more in the order of birth and having consumed antiparasitic drugs in the last 6 months (p<0.005) (Tables 2 and 3).

### Association between maternal employment status and stunting in children 6 to 36 months of age

In the unadjusted and adjusted analyses, no significant association was found between maternal employment status and the presence of child stunting PR(u) = 1.14 (95% CI: 1. 0–1.4), PR(a) = 1.04 (95% CI: 0.9–1.2), taking into account the multi-stage study design (Table 4).

**Table 4:** Association between mother’s work (Model 1) and the presence of stunting in children from 6 to 36 months of age after adjustment of both for control variables, taking into account the complex sample design.

Associated factors	Stunting		Unadjusted Model			Adjusted Model		
	Yes	(%)	PR(u)	95% CI	*P-value*	PR(a)	95% CI	*P-value*^a^
**Characteristics of mothers**								
*Age*^*b*^								
15–24 years	257	(14.8)	Ref.			Ref.		
25–34 years	375	(14.7)	0.99	0.81–1.22	0.94	0.87	0.71–1.07	0.198
35–49 years	235	(16.5)	1.11	0.89–1.39	0.37	0.91	0.71–1.15	0.422
*Level of education*^*a*^								
Without education	53	(49.2)	Ref.			Ref.		
Primary	409	(30.8)	0.63	0.49–0.80	**<0.001**	0.80	0.62–1.03	0.079
Secondary	380	(10.6)	0.22	0.17–0.28	**<0.001**	0.55	0.42–0.72	**<0.001**
Superior	25	(4.8)	0.10	0.06–0.17	**<0.001**	0.35	0.20–0.61	**<0.001**
*Marital Status*^*b*^								
Without partner	109	(13.0)	Ref.			Ref.		
With partner	758	(15.6)	1.20	0.93–1.55	0.162	0.96	0.76–1.23	0.766
*Economic level*^*b*^								
Very poor / poor	724	(25.3)	Ref.			Ref.		
Medium/ Rich / Very rich	143	(6.5)	0.26	0.20–0.33	**<0.001**	0.57	0.42–0.77	**<0.001**
*Region of Residence*^*b*^								
Coast	139	(6.9)	Ref.			Ref.		
Andes highland	452	(26.2)	3.81	2.97–4.90	**<0.001**	2.17	1.63–2.90	**<0.001**
Amazon basin	276	(22.8)	3.32	2.54–4.34	**<0.001**	1.67	1.25–2.23	**<0.001**
*Area of residence*^*b*^					**<0.001**			
Urban	318	(9.3)	Ref.			Ref.		
Rural	549	(30.3)	3.27	2.73–3.93	**<0.001**	1.22	0.99–1.51	0.068
*Number of children < 5 years of age*^*b*^								
1	394	(11.0)	Ref.			Ref.		
2	372	(20.5)	1.87	1.57–2.23	**<0.001**	1.41	1.20–1.67	**<0.001**
3+	101	(28.9)	2.63	1.98–3.50	**<0.001**	1.71	1.33–2.20	**<0.001**
***Employment status***^*b*^								
Working	257	(14.0)	Ref.			Ref.		
Not Working	610	(15.9)	1.14	0.95–1.37	0.167	1.04	0.88–1.23	0.627
**Characteristics of children**								
*Child Age*^*b*^								
06–12 months	139	(115)	Ref.			Ref.		
13–18 months	175	(15.8)	1.38	1.07–1.76	**0.014**	1.46	1.14–1.87	**0.002**
19–24 months	215	(18.7)	1.63	1.28–2.07	**<0.001**	1.55	1.24–1.94	**<0.001**
25–30 months	158	(13.9)	1.21	0.92–1.60	0.174	1.25	0.95–1.65	0.116
31–36 months	180	(16.7)	1.45	1.13–1.87	**0.004**	1.35	1.06–1.71	**0.014**
*Gender*^*b*^								
Male	505	(17.0)	Ref.			Ref.		
Female	362	(13.3)	0.78	0.66–0.91	**0.002**	0.75	0.65–0.86	**<0.001**
*Birth order*^*b*^								
First child	188	(9.2)	Ref.			Ref.		
2^nd^– 4^th^ child	460	(15.6)	1.70	1.38–2.09	**<0.001**	1.33	1.06–1.69	**0.016**
5^th^ child or greater	219	(33.2)	3.61	2.86–4.55	**<0.001**	1.51	1.11–2.06	**0.010**
*Consumption of antiparasitic drugs in the last 6 month*^*b*^								
No	585	(13.8)	ref			Ref.		
Yes	282	(19.7)	1.43	1.20–1.70	**<0**.**001**	1.18	1.00–1.39	0.056

^a^ Adjusted to mother’s age, educational level, marital status, economic level, region, place of residence, number of children under 5 years old, child age, child sex, birth order, current/antecedent condition of breastfeeding and antiparasitic drug consumption in the last six months.

^b^ Chi-squared test for categorical variables and Poisson regression in the unadjusted and adjusted model both with a 95% confidence interval taking into account the complex sample design: probabilistic, stratified, two-stage, independent, and self-weighted survey conducted by department.

### Association between type of remuneration and stunting in children 6 to 36 months of age

Although the association between maternal employment status and the presence of child stunting was not significant, the unadjusted and adjusted analyses showed that the type of remuneration received by the mothers had a significant association with the presence of stunting (Table 5). Mothers who work and are unpaid had a PR(u) = 2.95 (95% CI: 2.5–3.6) in the unadjusted analysis and a PR(a) = 1.38 (95% CI: 1.2–1.6) in the adjusted analysis compared with working mothers paid in money and/or in-kind, taking into account the multi-stage study design (Table 5).

**Table 5:** Association between the type of remuneration of working mothers (Model 2) and the presence of stunting in children from 6 to 36 months of age after adjustment of both for control variables, taking into account the complex sample design.

Associated factors	Stunting		Unadjusted Model			Adjusted Model		
	Yes	(%)	PR(u)	95% CI	*P-value*	PR(a)	95% CI	*P-value*^a^
**Characteristics of mothers**								
*Age*^*b*^								
15–24 years	257	(14.8)	Ref.			Ref.		
25–34 years	375	(14.7)	0.99	0.81–1.22	0.94	0.89	0.72–1.09	0.250
35–49 years	235	(16.5)	1.11	0.89–1.39	0.37	0.93	0.73–1.18	0.541
*Level of education*^*b*^								
Without education	53	(49.2)	Ref.			Ref.		
Primary	409	(30.8)	0.63	0.49–0.80	**<0.001**	0.81	0.63–1.05	0.106
Secondary	380	(10.6)	0.22	0.17–0.28	**<0.001**	0.57	0.43–0.75	**<0.001**
Superior	25	(4.8)	0.10	0.06–0.17	**<0.001**	0.37	0.22–0.65	**<0.001**
*Marital Status*^*b*^								
Without partner	109	(13.0)	Ref.			Ref.		
With partner	758	(15.6)	1.20	0.93–1.55	0.162	0.92	0.73–1.18	0.520
*Economic level*^*b*^								
Very poor / poor	724	(25.3)	Ref.			Ref.		
Medium/ Rich / Very rich	143	(6.5)	0.26	0.20–0.33	**<0.001**	0.56	0.41–0.77	**<0.001**
*Region of residence*^*b*^								
Coast	139	(6.9)	Ref.			Ref.		
Andes highland	452	(26.2)	3.81	2.97–4.90	**<0.001**	2.17	1.62–2.89	**<0.001**
Amazon basin	276	(22.8)	3.32	2.54–4.34	**<0.001**	1.61	1.20–2.15	**<0.001**
*Area of residence*^*b*^					**<0.001**			
Urban	318	(9.3)	Ref.			Ref.		
Rural	549	(30.3)	3.27	2.73–3.93	**<0.001**	1.16	0.93–1.44	0.018
*Number of children < 5 years of age*^*b*^								
1	394	(11.0)	Ref.			Ref.		
2	372	(20.5)	1.87	1.57–2.23	**<0.001**	1.42	1.20–1.68	**<0.001**
3+	101	(28.9)	2.63	1.98–3.50	**<0.001**	1.71	1.32–2.20	**<0.001**
***Type of remuneration***^*b*^								
Paid in money and/or in-kind	390	(12.7)	Ref.			Ref.		
Unpaid	220	(37.3)	2.95	2.45–3.55	**<0.001**	1.38	1.17–1.64	**<0.001**
Not working	257	(14.0)	1.10	0.91–1.34	0.322	1.05	0.87–1.26	0.614
**Characteristics of children**								
*Age of infant*^*b*^								
06–12 months	139	(115)	Ref.			Ref.		
13–18 months	175	(15.8)	1.38	1.07–1.76	**0.014**	1.46	1.14–1.86	**0.003**
19–24 months	215	(18.7)	1.63	1.28–2.07	**<0.001**	1.56	1.25–1.95	**<0.001**
25–30 months	158	(13.9)	1.21	0.92–1.60	0.174	1.26	0.96–1.66	0.098
31–36 months	180	(16.7)	1.45	1.13–1.87	**0.004**	1.36	1.07–1.72	**0.012**
*Gender of infant*^*b*^								
Male	505	(17.0)	Ref.			Ref.		
Female	362	(13.3)	0.78	0.66–0.91	**0.002**	0.75	0.66–0.86	**<0.001**
*Birth order*^*b*^								
First child	188	(9.2)	Ref.			Ref.		
2^nd^– 4^th^ child	460	(15.6)	1.70	1.38–2.09	**<0.001**	1.34	1.06–1.69	**0.015**
5^th^ child or greater	219	(33.2)	3.61	2.86–4.55	**<0.001**	1.48	1.08–2.02	**0.014**
*Consumption of antiparasitic drugs in the last 6 months*^*b*^								
No	585	(13.8)	ref			Ref.		
Yes	282	(19.7)	1.43	1.20–1.70	**<0**.**001**	1.17	0.99–1.39	0.063

a Adjusted to mother’s age, educational level, marital status, economic level, region, place of residence, number of children under 5 years old, child age, child sex, birth order, current/antecedent condition of breastfeeding and antiparasitic drug consumption in the last six months.

b Chi-squared test for categorical variables and Poisson regression in the unadjusted and adjusted model both with a 95% confidence interval taking into account the complex sample design: probabilistic, stratified, two-stage, independent, and self-weighted survey conducted by department.

It was also shown that mothers who worked and did not receive any remuneration were mostly those who worked for a family member (82.1%).

Table 6 describes the socio-demographic characteristics of the mothers with children between 6 to 36 months of age according to the type of remuneration. Moreover, to more accurately describe the significant association between extreme poverty and unpaid work and stunting we used the original Wealth Index of the DHS-Peru that consists in five levels (very poor, poor, medium, rich, very rich).

**Table 6:** Socio-demographic characteristics of mothers with children between 6 to 36 months of age according to the type of remuneration (N = 4637).

Characteristics of mothers	Type of remuneration						
	Paid in money and/or in-kind		Unpaid		Not working		P-value
	(n 2475)		(n 576)		(n 1586)		
	n	(%)	n	(%)	n	(%)	
*Age*^*a*^							**<0.001**
15–24 years	636	(25.6)	187	(33.2)	588	(36.5)	
25–34 years	1181	(47.0)	248	(42.3)	683	(42.4)	
35–49 years	658	(27.4)	141	(24.5)	315	(21.1)	
*Level of education*^*a*^							**<0.001**
Without education	42	(1.2)	36	(6.0)	35	(2.0)	
Primary	519	(17.4)	311	(51.7)	414	(22.2)	
Secondary	1572	(67.0)	223	(41.2)	1039	(68.9)	
Superior	342	(14.4)	6	(1.1)	98	(6.9)	
*Marital status*^*a*^							**<0.001**
Without partner	460	(18.2)	38	(9.2)	122	(8.3)	
With partner	2015	(81.8)	538	(90.8)	1464	(91.7)	
*Economic level*^*a*^							**<0.001**
Very poor	575	(15.8)	438	(70.5)	451	(22.3)	
Poor	692	(23.9)	77	(13.0)	497	(26.0)	
Medium	510	(23.3)	34	(7.6)	318	(22.7)	
Rich	392	(20.4)	19	(5.8)	210	(17.7)	
Very rich	306	(16.6)	8	(3.1)	110	(11.3)	
*Region of residence*^*a*^							**<0.001**
Coast	1019	(56.4)	48	(15.1)	698	(59.3)	
Andes highland	898	(30.0)	292	(47.6)	468	(25.2)	
Amazon basin	558	(13.6)	236	(37.3)	420	(15.5)	
*Area of residence*^*a*^							**<0.001**
Urban	1668	(77.3)	104	(24.3)	1036	(74.2)	
Rural	807	(22.7)	472	(75.7)	550	(25.8)	
*Number of Children < 5 years of age*^*a*^							**<0.005**
1	1159	(63.7)	296	(53.1)	901	(59.9)	
2	768	(30.3)	208	(35.1)	564	(33.8)	
3+	148	(6.0)	72	(11.8)	121	(6.3)	

^*a*^Chi-squared test for categorical variables

It was found that 42.3% of unpaid mothers are mainly between 25 to 34 years old, 51.7% had completed only primary education, 90.8% lived with their partners, 83.5% were poor to very poor, 47.6% inhabited the Andes highland region, 75.7% lived in a rural setting and most (53.1%) only had one child. It should be noted that on classifying the economic level into 5 levels we observed that 70.5% of the mothers who worked without being paid were from the lower wealth quintile, and 56.5% only had a primary education.

Finally, Table 7 compares the presence of stunting in children from 6 to 36 months with the two models under study, maternal employment status and type of remuneration.

**Table 7:** Comparison between mother’s work (Model 1), type of remuneration of working mothers (Model 2) and the presence of stunting in children from 6 to 36 months of age after adjustment of both for control variables, taking into account the complex sample design.

Associated factors	Stunting		Unadjusted Model			Adjusted Model		
	Yes	(%)	PR(u)	95% CI	*P-value*	PR(a)	95% CI	*P-value*^a^
***MODEL 1***								
***Work***^***b***^								
Not working	257	(14.0)	Ref.			Ref.		
Working	610	(15.9)	1.14	0.95–1.37	0.167	1.04	0.88–1.23	0.627
***MODEL 2***								
***Type of remuneration***^***b***^								
Paid in money and/or in-kind	390	(12.7)	Ref.			Ref.		
Unpaid	220	(37.3)	2.95	2.45–3.55	**<0.001**	1.38	1.17–1.64	**<0.001**
Not working	257	(14.0)	1.10	0.91–1.34	0.322	1.05	0.87–1.26	0.614

^a^ Adjusted to mother’s age, educational level, marital status, socioeconomic level, region, place of residence, number of children under 5 years-old, child age, child sex, birth order, current/antecedent condition of breastfeeding and antiparasitic drug consumption in the last six months.

^b^ Chi^2^ test for categorical variables and Poisson regression in the unadjusted and adjusted model both with 95% confidence interval taking into account the complex sample design: probabilistic, stratified, two-stage, independent, and self-weighted survey conducted by department.

## Discussion

### Main findings

In this population-based study, the prevalence of stunting in children from 6 to 36 months of age was 15.9% (95% CI: 13.9–16.7). No significant association was found between the employment status of the mother and the presence of child stunting in the unadjusted and adjusted analyses. On the other hand, we found that unpaid mothers had a higher prevalence of children with stunting than those who work and are paid in money and/or in-kind.

### Comparison with other studies

The prevalence of stunting in Peru has been decreasing annually, indicating an improvement in the nutritional status of the children. Indeed, in the last 14 years, the percentage of stunting decreased from 31.0% to 14.6%[6]. Stunting in the first stages of life has an important impact on child growth and development. Although no association was found between maternal employment status (working and non-working), there was a significant association between the type of remuneration of working mothers and the nutritional status of their children, suggesting that a higher income can provide children with a better quality of life.

While the association between child stunting and maternal employment status was not significant in the present analysis, some publications have shown a lower prevalence of stunting among children of working mothers. A previous study carried out in a rural area of Peru found that children of non-working mothers had a higher prevalence of stunting than those of paid working mothers (p<0.001)[20]. Moreover, a Colombian study found that the prevalence of stunting in children of working mothers was lower compared with non-working mothers (p = 0.012)[19]. Furthermore, Asian and African studies have found that the prevalence of stunted children is higher in those of non-working mothers than of those of working mothers (p<0.01)[17,18,23], likely because the latter have greater access to and acquire better sources of food for their children [17–20,23].

In contrast, in a Latin-American study of a Brazilian suburb, a higher prevalence of stunting was found among children in the groups of working mothers compared with non-working mothers (p = 0.04)[14]. Likewise, in two Indian studies, working mothers had a higher prevalence of stunted children than non-working mothers (p<0.05)[15,24], similar to what was described in an Ethiopian study (p<0.001)[16]. These results are due to the lack of time that working mothers have to breastfeed, feed and look after their children[14–16,24–25].

Before the 21st century, women did not usually work outside the home, and therefore, did not participate actively in bringing external resources to the household economy. Nowadays, women have entered the labour market for several reasons; some for personal achievement or in order to have an independent income, while others work due to the necessity of raising the economic and social status of their families. In addition, a better economic status allows mothers to obtain access to better sources of food for themselves and their children[20]. Nonetheless, working can decrease the time mothers have to breastfeed, feed and look after their children. Some studies have suggested that in many developing countries poor women have multiple roles in family income-generating activities which, in many cases, reduces childcare time, affecting the nutritional status of the children[26–28].

An important fact found in our study is that 82.1% of the unpaid working mothers works for their family members. These women work to help their families doing agricultural or other activities with no monetary or in-kind payment which lead to an increase in the prevalence of children with stunting as found in our study. This may be due to the lack of time unpaid working mothers have to care for their children and a lack of money and/or food to adequately feed them.

Other important characteristics of unpaid working mothers are that 75.7% lived in a rural setting, 47.6% inhabited the Andes highland and 37.3% in the amazon basin. It should be noted that the majority of mothers who worked in those areas did not have a formal employment controlled by labour laws.

As an example, Peru has the law No. 29896 that establishes the implementation of breastfeeding in the public and private sector institutions promoting an hour of breastfeeding during the first 24 months of the child’s life. Moreover, a mandatory 98-day maternity leave is established by the law No. 30367. Unfortunately, both of these laws are not followed in the absence of a formal job. This situation occurs constantly in small cities or rural areas where mothers work for small business. There exists a Peruvian law on equal opportunities for women and men; however this is rarely applied in rural areas far away from the main cities. Due to these inequalities, marches against discrimination against women are increasingly being carried out in our country.

Finally, as in other studies, some of our co-variables showed a significant association between stunting and confounding variables such as “no education” compared to mothers with education[16–17,23,25,28–37]. In addition, belonging to a “very poor” economic status is associated with the presence of stunting compared to a “medium”, “rich” or “very rich” economic status[14,16,19,20,23,31–34,36,38]. Compared to residing on the “coast”, residing in the “Andes highland” is associated with the presence of stunting[37], and residing in “rural” areas increases the prevalence of stunting compared to residing in “urban” areas[16,20,32,38–41]. Furthermore, having 3 or more children at home[14,31,37,39] and being the fifth or more in the order of birth, increases the prevalence of stunting[16,30,34–35,37–38]. All of these variables were independently associated with the outcome in our analyses. As found in other studies with similar results, these associations contribute to the validity of our analysis.

### Strengths and limitations

Among the strengths of the present study, it should be highlighted that this was a population-based study, including a representative nationwide sample. Likewise, the data of the DHS-Peru was collected by trained personnel, ensuring reliable results. Adjustment of the different weighted control variables to a multistage study, provided the most accurate results about a possible association between the exposure and outcome variables.

Nonetheless, our study has some limitations. Since it was based on a secondary source we included some variables that were not evaluated in the DHS-Peru, but which were considered in other studies, such as the working hours of the mother, the actual amount of remuneration, family income and knowledge of the mother about nutrition. There are also other variables with incomplete data that could not be included in our analyses, such as who is the caregiver of the child, who feeds the child, the presence of fever, diarrhoea and acute respiratory infections in the last 2 weeks, among others. However, their distribution may not present bias due to poor classification. Nevertheless, a previous analysis with these variables did not alter the results of the study, and therefore, they are unlikely to present a bias.

Moreover, self-reported data collection was used in the DHS-Peru, which may have produced an information bias with errors in the information provided by the mothers due to a lack of memory.

### Implications

The high prevalence of stunting among infants of unpaid working mothers suggests the need to implement educational programs on the possible consequences of unpaid work and how these could impact in the nutritional status of children. Several studies provide evidence about the effectiveness of pregnancy counseling in the improvement of quality of life for mothers and children through primary prevention[42]. It is important to provide counselling before and during pregnancy to all women about the importance of an adequate nutrition and supplementation as well as about childcare during the early years with the aim of decreasing the high prevalence of stunting, which carries several repercussions in adulthood[42–44]. Moreover, educating mothers during pregnancy is associated with positive maternal behaviors that could reduce the amount of unpaid work which has been associated with the presence of stunting in the present study[43]. Finally, adequate labour policies are also needed to ensure a greater number of paid jobs for this population in order to reduce the prevalence of stunting among children. These laws should be monitored to ensure that they are enforced or executed. Further studies would confirm the findings of an association presented here.

## Conclusions

No significant association was found between maternal employment status and the presence of stunting in children 6 to 36 months of age. However, the prevalence of child stunting increased among infants of unpaid working mothers compared with those of mothers who are paid with money and/or in-kind.
