# Correction: Relationship between stunting in children 6 to 36 months of age and maternal employment status in Peru: A sub-analysis of the Peruvian Demographic and Health Survey

**DOI:** 10.1371/journal.pone.0217252

**Published:** 2019-06-11

**Authors:** Airin Chávez-Zárate, Jorge L. Maguiña, Antoinette Danciana Quichiz-Lara, Patricia Edith Zapata-Fajardo, Percy Mayta-Tristán

There are errors in the author affiliations. The correct affiliations are as follows:

Airin Chávez-Zárate^1^, Jorge L. Maguiña^2,3^, Antoinette Danciana Quichiz-Lara^1^, Patricia Edith Zapata-Fajardo^1^, Percy Mayta-Tristán^2,3,4^

**1** Escuela de Nutrición y Dietética, Universidad Peruana de Ciencias Aplicadas, Lima, Perú, **2** Escuela de Medicina, Universidad Peruana de Ciencias Aplicadas, Lima, Perú, **3** Facultad de Ciencias de la Salud, Universidad Científica del Sur, Lima, Perú, **4** Dirección General de Investigación, Desarrollo e Innovación, Universidad Científica del Sur, Lima, Perú.

The last author’s email address is also incorrect. Dr. Mayta-Tristán’s correct email address is: pmayta@cientifica.edu.pe.
